# Neutrophils in Cancer immunotherapy: friends or foes?

**DOI:** 10.1186/s12943-024-02004-z

**Published:** 2024-05-18

**Authors:** Xueqin Huang, Eugenie Nepovimova, Vojtech Adam, Ladislav Sivak, Zbynek Heger, Marian Valko, Qinghua Wu, Kamil Kuca

**Affiliations:** 1 College of Life Science, Yangtze University, Jingzhou, 434025 China; 2 Department of Chemistry, Faculty of Science, University of Hradec Králové, 500 03 Hradec Králové, Czech Republic; 3 Department of Chemistry and Biochemistry, Mendel University in Brno, 613 00 Brno, Czech Republic; 4 Faculty of Chemical and Food Technology, Slovak University of Technology, 812 37 Bratislava, Slovakia; 5 Biomedical Research Center, University Hospital Hradec Kralove, 500 05 Hradec Kralove, Czech Republic; 6 Andalusian Research Institute in Data Science and Computational Intelligence (DaSCI), University of Granada, Granada, Spain

**Keywords:** Neutrophils, Cancer, Immunotherapy, Antitumor activity, Protumor activity

## Abstract

Neutrophils play a Janus-faced role in the complex landscape of cancer pathogenesis and immunotherapy. As immune defense cells, neutrophils release toxic substances, including reactive oxygen species and matrix metalloproteinase 9, within the tumor microenvironment. They also modulate the expression of tumor necrosis factor-related apoptosis-inducing ligand and Fas ligand, augmenting their capacity to induce tumor cell apoptosis. Their involvement in antitumor immune regulation synergistically activates a network of immune cells, bolstering anticancer effects. Paradoxically, neutrophils can succumb to the influence of tumors, triggering signaling cascades such as JAK/STAT, which deactivate the immune system network, thereby promoting immune evasion by malignant cells. Additionally, neutrophil granular constituents, such as neutrophil elastase and vascular endothelial growth factor, intricately fuel tumor cell proliferation, metastasis, and angiogenesis. Understanding the mechanisms that guide neutrophils to collaborate with other immune cells for comprehensive tumor eradication is crucial to enhancing the efficacy of cancer therapeutics. In this review, we illuminate the underlying mechanisms governing neutrophil-mediated support or inhibition of tumor progression, with a particular focus on elucidating the internal and external factors that influence neutrophil polarization. We provide an overview of recent advances in clinical research regarding the involvement of neutrophils in cancer therapy. Moreover, the future prospects and limitations of neutrophil research are discussed, aiming to provide fresh insights for the development of innovative cancer treatment strategies targeting neutrophils.

## Introduction

Neutrophils, the most abundant population of white blood cells in circulation, play critical roles in host defense against infection [1]. Traditionally recognized as inflammatory immune cells, neutrophils eliminate pathogens through phagocytosis, degranulation, and neutrophil extracellular trap (NET) formation [2]. Under normal conditions, neutrophils have a brief lifespan, and their activation and mobilization are tightly controlled by the body to prevent potential harm to normal tissues from the highly toxic nature of neutrophil responses [3]. However, in pathological settings, the continuous infiltration of neutrophils can lead to chronic inflammation and tissue damage, which often contribute to tumorigenesis [4]. Recent research has elucidated the multifaceted nature of neutrophils, revealing that they not only serve as effective antibacterial cells but also exhibit phenotypic and functional heterogeneity in pathological contexts such as cancer [5, 6]. Consequently, the impact of neutrophils on tumors has emerged as a prominent area of investigation.

In this work, we present a comprehensive overview of recent research advancements aimed at unraveling the dual mechanisms of neutrophils in tumor pathogenesis and their potential in immunotherapy. We specifically focus on various factors influencing the phenotypic alterations of tumor-associated neutrophils and predict their clinical utility as diagnostic tools and vital targets for antitumor interventions. Additionally, we explore the existing opportunities and challenges in neutrophil research. This work enhances our understanding of the role of neutrophils in cancer and offers systematic insights for the development of cancer immunotherapy strategies targeting neutrophils.

## Neutrophils and cancer

**Fig. 1:**
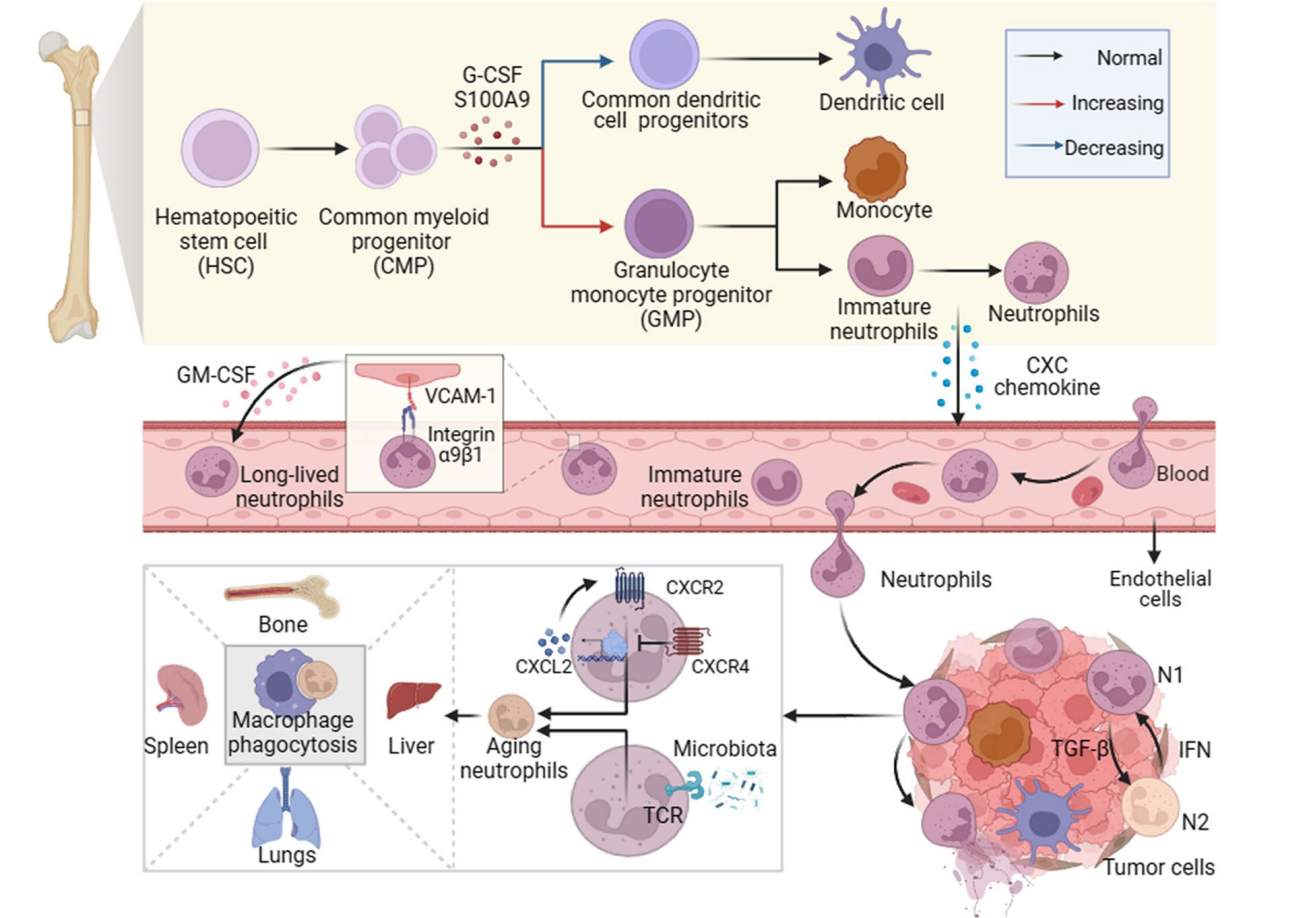
The development, mobilization, and clearance of neutrophils in the tumor microenvironment. Hematopoietic progenitor stem cells (HSCs) in the bone marrow differentiate into common myeloid progenitor cells (CMPs), which give rise to granulocyte-monocyte progenitor cells (GMPs) and eventually mature segmented neutrophils. Tumor-derived mediators such as granulocyte colony-stimulating factor (G-CSF) and S100 calcium-binding protein A9 (S100A9) promote the differentiation of the neutrophil and monocyte lineages while leading to systemic dendritic cell deficiency in vivo. Chemokines trigger the mobilization of mature and immature neutrophils into the circulation. Immature neutrophils, known as polymorphonuclear myeloid-derived suppressor cells (PMN-MDSCs), are considered in this context. During transendothelial migration, the interaction between integrin α9β1 on neutrophils and vascular cell adhesion molecule 1 (VCAM-1) on endothelial cells stimulates the release of granulocyte-macrophage colony-stimulating factor (GM-CSF) from the latter, prolonging neutrophil lifespan. Neutrophils that extravasate into the tumor tissue adopt antitumor (type N1) or protumor (type N2) phenotypes, influenced by growth factor-β (TGF-β) and type 1 interferon (IFN), respectively. After fulfilling their functions, neutrophils undergo senescence due to intrinsic programs (CXCR2 or CXCR4) and extrinsic factors (microbiota) and are subsequently cleared by macrophages in the bone marrow, spleen, liver, and lungs. Neutrophils also undergo programmed death to form neutrophil extracellular traps (NETs)

Neutrophils are derived from hematopoietic stem cells (HSCs) located in the bone marrow. The process begins with the differentiation of HSCs into common myeloid progenitors (CMPs), which further develop into granulocyte monocyte progenitors (GMPs) and eventually give rise to mature segmented neutrophils [7]. During carcinogenesis, the hematopoietic system may tend to produce more neutrophils and monocytes [8]. This shift in differentiation is driven by the upregulation of granulocyte colony-stimulating factor (G-CSF) and myeloid-related protein S100 calcium-binding protein A9 (S100A9), inhibiting dendritic cell differentiation while promoting the accumulation of bone marrow-derived suppressor cells [9, 10]. This skewed differentiation process further facilitates immune evasion by tumors, thereby promoting tumor progression and treatment resistance (Fig. 1). Of particular intrigue, polymorphonuclear-myeloid-derived suppressor cells (PMN-MDSCs), exhibit protumor activity akin to that of immunosuppressive neutrophils [11]. Under the stimulation of granulocyte colony-stimulating factor (G-CSF), GM-CSF, pro-inflammatory cytokines interleukin-6 (IL-6), IL-8, TGF-β, and leukotriene B4, immature PMN-MDSCs undergo massive expansion from their precursors in the bone marrow and venture into the peripheral blood and tumor microenvironment [12–17]. While distinguishing PMN-MDSCs from regular neutrophils in mice remains challenging, distinct markers have been identified for human PMN-MDSCs [18]. The biomarkers/features related to classic neutrophils and PMN MDSCs are shown in Table 1.

**Table 1:** Comparison of basic features of classical neutrophils and polymorphonuclear myeloid derived suppressor cells (PMN-MDSCs)

Characteristic	Neutrophils	PMN-MDSCs	References
Activation effects	Host infection with bacteria or pathogens	Long-term chronic inflammation, autoimmune diseases and cancer	[11]
Mutagenic factors	G-CSF, GM-CSF	G-CSF, GM-CSF, IL-6, IL-8, TGF-β, leukotriene B4	[12–17, 19, 20]
Cell surface marker	CD14^−^CD15^+^CD66b^+^CD16^+^	CD14^−^CD15^+^CD66b^+^CD16^+^CD11b^+^CD33^+^HLA-DR^−^	[21–24]
New specific biomarkers	c-Kit^hi^CXCR2^hi^CD101^hi^	LOX1^hi^FATP2^hi^Arg-1^hi^CD300ld^hi^	[25–30]
Nuclear morphology	Segmented nucleus	Banded nucleus	[31]
Mature state	Mature	Immaturate	[31]
Density centrifugation	High-density cells	Low-density cells	[31]

Factors like G-CSF and GM-CSF regulate neutrophil development, prompting the release of mature neutrophils from the bone marrow [19, 20]. However, sustained chronic inflammation, autoimmune ailments, and the provocation of cancer may result in neutrophil depletion, setting off an occurrence known as “emergency granulopoiesis,” rapidly facilitating the influx of mature or even immature neutrophils into the circulation [11, 32]. As neutrophils age within the body, they depart from their target sites and are subsequently cleared by macrophages in the bone marrow, spleen, liver, and lungs [33]. Interestingly, both intrinsic and extrinsic factors influence neutrophil aging. Brain and muscle Arnt-like 1 (BMAL1) drives cell aging by inducing the production of CXCL2 and subsequent CXCR2 signaling, while CXCR4 antagonizes this aging effect [34]. Moreover, the microbiome influences neutrophil senescence through a signaling pathway mediated by toll-like receptors and myeloid differentiation factor 88 (MYD88), representing an external mode of regulation [35]. During transendothelial cell migration, the interaction between integrin α9β1 on neutrophils and vascular cell adhesion molecule 1 (VCAM-1) on endothelial cells triggers the release of GM-CSF, thereby prolonging neutrophil survival [36]. Neutrophils also undergo a distinct form of cell death known as NETosis, wherein they release their nuclear DNA and lytic proteins into the extracellular space in the form of NETs [37]. Neutrophils have considerable plasticity and complexity in the tumor microenvironment. The transforming growth factor-β (TGF-β) and type I interferons (IFN 1) respectively induce the polarization of neutrophils towards the N2 and N1 phenotypes [16, 38] (Fig. 1). In the circulation of cancer patients and mice, high-density neutrophils (HDNs) are a uniform group of mature (segmented) neutrophils with anti-tumor properties, while low-density neutrophils (LDNs) are a mix of immaturce (banded) and mature neutrophils with immunosuppressive properties [31].

Therefore, tumor cells have significant regulatory effects on neutrophil recruitment, clearance, and polarization. There is confusion in naming cells with similar or overlapping mechanisms of action, such as PMN-MDSC and N2 neutrophils. Hence, it is necessary to further explore a more appropriate and comprehensive nomenclature.

## Anti-tumor effects of neutrophils

**Fig. 2:**
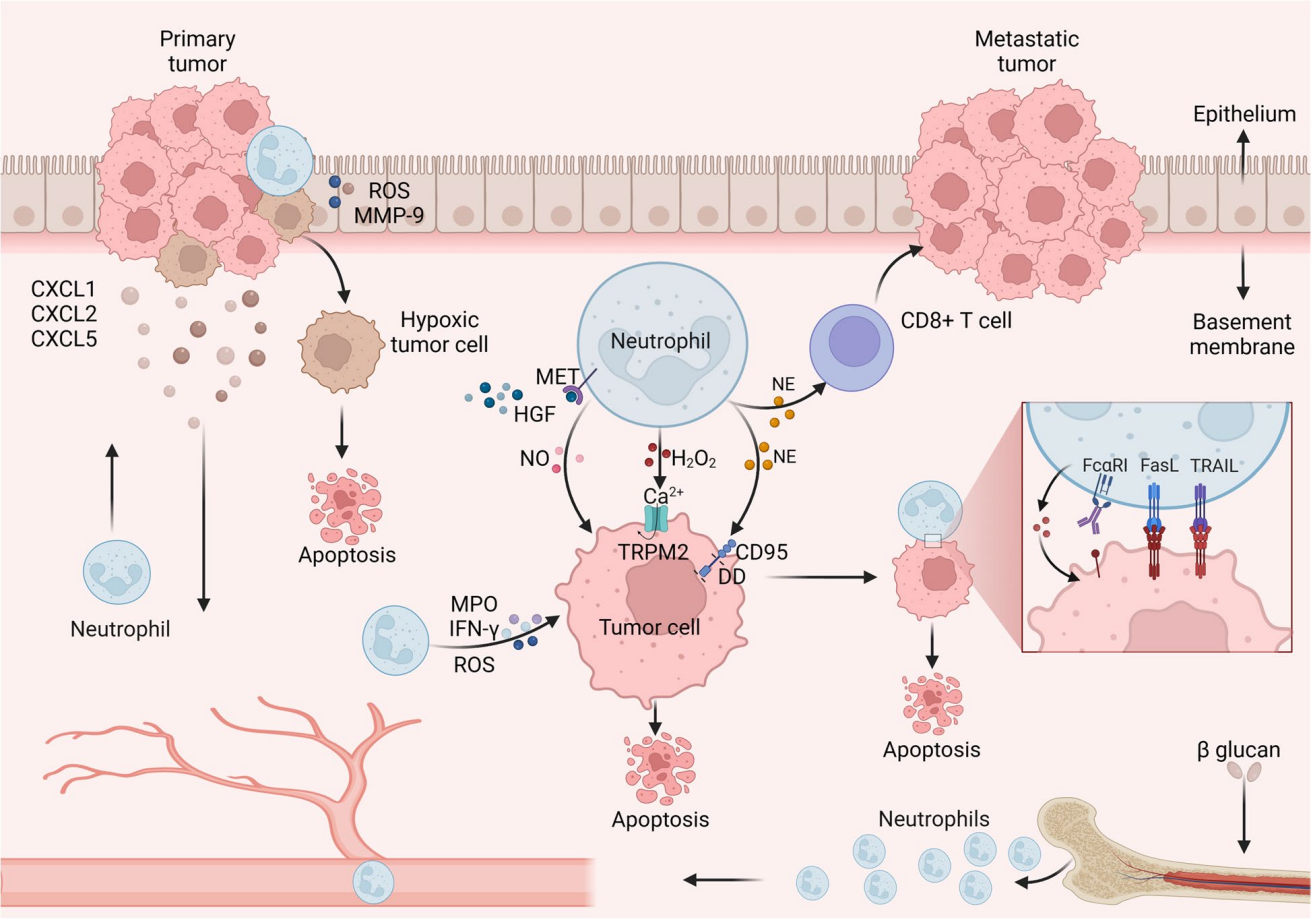
Direct cytotoxic effects of neutrophils on cancer cells. Neutrophils, when exposed to β-glucan, rely on the memory of bone marrow precursors and exert antitumor effects. The chemokines CXCL1, CXCL2, and CXCL5, secreted by primary tumors, facilitate the recruitment of neutrophils to the tumor site. Under hypoxic conditions, activated neutrophils induce reactive oxygen species (ROS) and matrix metalloproteinase (MMP-9) degradation of the epithelial basement membrane, which ultimately restricts tumor development. Neutrophils directly secrete ROS, myeloperoxidase (MPO), and interferon γ (IFN-γ) to inhibit tumor progression. The interaction between the ligand hepatocyte growth factor (HGF) and receptor tyrosine protein kinase (MET) on neutrophils leads to the release of nitric oxide (NO) by MET^+^ neutrophils, which exerts antitumor effects. Neutrophils secrete hydrogen peroxide (H_2_O_2_), inducing apoptosis in tumor cells through Ca2^+^ influx via the transient receptor potential cation channel, subfamily M, member 2 (TRPM2). Neutrophil elastase (NE) hydrolyzes and releases the CD95 death structure domain (DD), selectively killing cancer cells. Additionally, NE has distant effects on CD8^+^ T cells. Neutrophils with enhanced expression of TNF-related apoptosis-inducing ligand (TRAIL) and Fas ligand (FasL) induce apoptosis in cancer cells through direct contact

Neutrophils secrete various toxic factors or directly contact tumor cells to exert anti-tumor activity. In a mouse model of uterine cancer, the response to low oxygen levels attracted neutrophils to the tumor area [39]. These neutrophils then induced the separation of tumor cells from the basement membrane, thereby limiting the growth of early tumors [39]. Recent research has illuminated a captivating phenomenon: although reducing the hypoxic tumor environment may lead to fewer neutrophils being recruited, their ability to kill cells is exceptional [40]. This remarkable observation can be attributed to the secretion of reactive oxygen species (ROS) and matrix metalloproteinases (MMP-9) by activated neutrophils, leading to the degradation of the epithelial basement membrane [40]. Additionally, neutrophils treated with β-glucan rely on high ROS levels to fight tumors, a process linked to the development of neutrophil precursors’ memory in the bone marrow of trained mice [41]. Neutrophils secrete hydrogen peroxide (H_2_O_2_), which induces apoptosis in tumor cells through the influx of calcium ions (Ca^2+^) mediated by the transient receptor potential cation channel, subfamily M, member 2 (TRPM2) [42]. Notably, neutrophil elastase (NE) secreted by neutrophils can hydrolyze and release the CD95 death domain (DD), selectively killing cancer cells and minimizing toxicity to noncancer cells [43]. NE also induces distant effects mediated by CD8^+^ T cells [43]. Inflammatory modulators such as TNF-α induce the expression of MET in neutrophils. When activated by ligand hepatocyte growth factor (HGF), MET^+^ neutrophils release nitric oxide (NO) to limit tumor growth and metastasis [44]. Neutrophils directly engage with tumor cells to exert their antitumor activity. For instance, neutrophils with enhanced expression of tumor necrosis factor-related apoptosis-inducing ligand (TRAIL) and Fas ligand (FasL) induce apoptosis through direct contact with cancer cells [45, 46]. IL-17 has been shown to increase the expression of TRAIL as well as ROS, and IFN-γ to bolster the direct killing ability of neutrophils [47]. Moreover, antibody-dependent cytotoxicity (ADCC), triggered by the binding of the Fc region of monoclonal antibodies (mAbs) to activated Fc receptors (FcRs) on neutrophils, can exhibit strong antitumor effects [48] (Fig. 2).

**Fig. 3:**
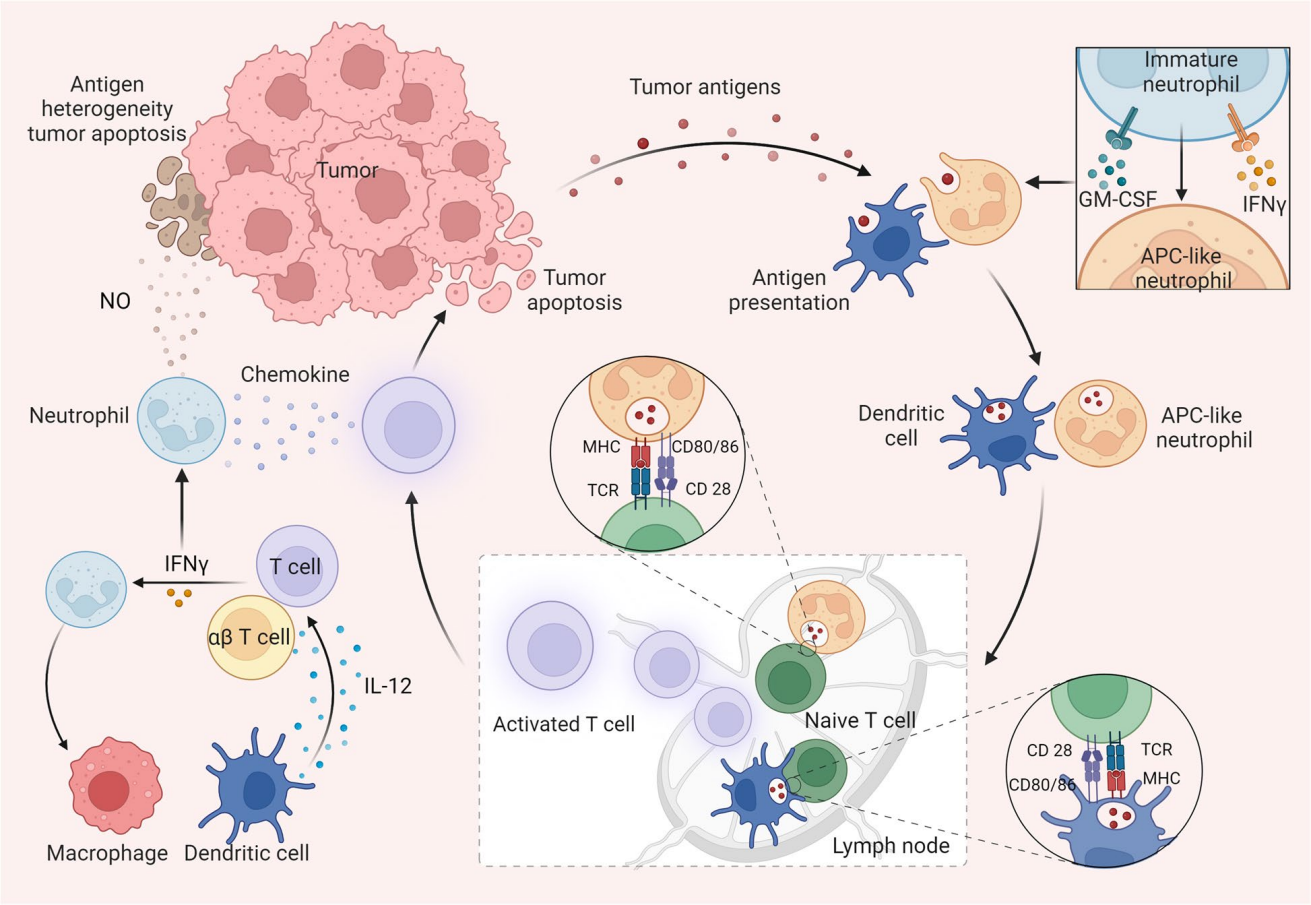
Neutrophils regulate immune cells to drive antitumor immune responses. When exposed to IFN-γ and GM-CSF, immature neutrophils can differentiate into hybrid neutrophils with antigen-presenting cell (APC) characteristics. Thereafter, dendritic cells and APC-like neutrophils can pick up tumor antigens and migrate to lymph nodes (LNs). In LNs, these antigen-presenting cells cross-present tumor antigens to T cells using MHC molecules and costimulatory ligands (CD80/CD86 on dendritic cells and APC-like neutrophils and CD28 on T cells), thereby stimulating antitumor T cell responses. Activated T cells then exit the LN and specifically target and eliminate tumor cells. Tumor cells can undergo antigenic variations, leading to the formation of tumor variants with diverse antigenic profiles. In response, activated T cells can secrete chemokines to recruit neutrophils, which contribute to the elimination of antigenically heterogeneous tumors by releasing NO. Interferons play critical roles in inducing antitumor immune responses in neutrophils. IL-12, secreted by dendritic cells and macrophages, triggers the type I activation of T cells and αβ T cells, resulting in the production of IFN-γ. Subsequently, activated neutrophils can further stimulate macrophages to release IL-12, amplifying the antitumor immune response

Neutrophils also play crucial roles in activating and regulating both innate and adaptive immunity. Recent findings suggest that T cells perform immune surveillance by recognizing specific antigens expressed by tumor cells. Simultaneously, activated T cells signal neutrophils to eliminate tumor escape variants with antigen heterogeneity [49]. Therefore, neutrophils are indispensable for the complete eradication of tumors, and this effect is partially dependent on iNOS [49]. Early research discovered that under the influence of IFN-γ and GM-CSF, immature neutrophils in the tumor microenvironment differentiate into hybrid neutrophils with antigen-presenting cell (APC) characteristics to stimulate anticancer T-cell responses [50]. Subsequent studies have explained more details of this process. During the pretransfer stage, APC-like neutrophils capture tumor antigens, migrate to the tumor-draining lymph nodes (LNs), and form synapses with T cells, presenting antigens to T cells and triggering antitumor immune responses [51]. Recent research shows that successful immunotherapy triggers a notable increase in tumor-infiltrating neutrophils, defining a distinct antitumor immune state termed the *Sell*^*hi*^ state [52]. The production of IL-12, a crucial cytokine, primarily relies on dendritic cells (DCs) and macrophages. Upon IL-12 activation, T cells release IFNγ, which triggers the expression of interferon response transcription factor (IRF1) in neutrophils, thereby exerting antitumor effects [52]. In turn, antitumor neutrophils further drive macrophages to release IL-12, which promotes unconventional αβ T cell type I activation and IFN-γ production, leading to the inhibition of sarcoma progression in mice [53] (Fig. 3).

Neutrophils express a wide range of cytotoxic factors or apoptosis-related ligands that mediate direct killing effects on tumors. Since neutrophils have a short lifespan, this immune response could be transient. However, neutrophil interactions with other components of the immune system induce long-term adaptive immune responses (refer to Table 2 for details).

**Table 2:** The antitumor mechanisms of neutrophils in different cancer models

Cancer type	Model	Influence factor	Effect	Anticancer mechanism	Reference
Uterine cancer	PTEN-deficient uterine cancer mice	Hypoxia	Relieving hypoxia decreases neutrophil recruitment while enhancing their cytotoxic effect.	Neutrophils induce ROS and MMP-9 to break down the epithelial basement membrane, which slows the growth of tumors.	[39, 40]
Melanoma	B16-F10 tumor–bearing mice	β-glucan	Neutrophils rely on ROS production to exert antitumor activity.	β-glucan induces neutrophil reprogramming into an antitumor phenotype, which depends on the memory of neutrophil precursors in the bone marrow.	[41]
Breast cancer	4 T1 tumor–bearing mice and MDA-MB-231 cells lines	H_2_O_2_	Ca^2+^ lethal influx kills disseminated tumor cells.	H_2_O_2_ secreted by neutrophils induces Ca^2+^ influx into tumor cells through TRPM2.	[42]
TNBC, lung cancer, and melanoma	PDX model mice and genetically engineered mice	NE	NE selectively kills tumor cells and induces distant effects mediated by CD8^+^ T cells.	The hydrolysis of NE secreted by neutrophils releases the CD95 death domain, selectively killing cancer cells.	[43]
Lung cancer, fibrosarcomas, melanomas, and breast tumors	LLC lung cancer mice, T241 and B16F10 tumor–bearing mice, MMTV-PyMT+ transgenic mice, c-Myc-driven hepatocellular carcinomas in mice, and chemically induced colorectal cancers in mice	HGF-MET axis	Neutrophils release NO through the HGF/MET-dependent pathway to exert a killing effect on tumor cells.	HGF activates MET^+^ neutrophils to kill tumor cells by releasing NO.	[44]
Human acute T-lymphocytic leukemia/lung cancer, human epidermoid carcinoma, cervical cancer, and hepatocellular carcinoma	Jurkat cells/A549, A431, HeLa, and HepG2 cell lines	TRAIL/FasL	Neutrophils regulate the expression of their own apoptotic-related ligands to induce tumor cell apoptosis.	Neutrophils expressing TRAIL and FasL induce apoptosis of tumor cells in contact.	[45, 46]
Esophageal squamous cell carcinoma	ESCC cell lines (EC109 and KYSE30) and xenograft nude mouse models	IL-17	IL-17 recruits neutrophils and enhances their antitumor activity.	IL-17 stimulates tumor cells to release CXC chemokines, and promotes TTRAIL, ROS, MPO, and IFN-γ expression in neutrophils.	[47]
Melanoma	Human samples and B16: B78H1 tumor-bearing mice	T cell immunotherapies	Neutrophils are activated to completely eradicate tumors.	CD4^+^T cell therapy co-activated with OX40 or combined with CTLA-4 blocker activate neutrophils to eliminate antigenic heterogeneity in tumors.	[49]
Lung cancer	Lung tumor tissue	IFN-γ and GM-CSF	APC-like neutrophil formation stimulates T cell responses in early lung tumors.	Immature neutrophils downregulate the transcription factor Ikaros under the influence of IFN-γ and GM-CSF and thereafter differentiate into APC-like neutrophils for T cell activation.	[50]
Lung adenocarcinoma, colon cancer	Mice injected with the G12D Kras mutation carrying oncogene and lacking P53 tumor cells and MC38 tumor–bearing mice	Macrophages and DC and T cells	The expression of IRF1 in neutrophils induces an antitumor immune response.	DCs and macrophages are producers of IL-12, which activates T cells to secrete IFNγ, leading to the induction of IRF1 expression in neutrophils.	[52]
Sarcoma	3-MCA-induced sarcoma mice	Neutrophils and macrophages	IFNγ is produced by unconventional αβ T cells.	Neutrophils drive macrophages to release IL-12, which promotes IFN-γ production by unconventional αβ T cells.	[53]

## Pro-tumor effect of neutrophils

Neutrophils, often drive tumor progression by fostering local tumor initiation and proliferation, promoting angiogenesis, facilitating tumor metastasis, and orchestrating networks of immune suppression within the tumor microenvironment (refer to Table 3 for details).

**Table 3:** The protumor mechanisms of neutrophils in different cancer models

Cancer type	Model	Influence factor	Effect	Anticancer mechanism	Reference
Colorectal cancer	Recombinase-activating gene-2-deficient (Rag2^−/−^) mice	*Helicobacter pylori*	Infection leads to the accumulation of neutrophils in the colon, ultimately promoting intestinal carcinogenesis.	During infectious conditions, TNF-α-driven accumulation of neutrophils in the colon results in the release of abundant NO, promoting intestinal carcinogenesis.	[54]
Colon cancer	Gpx4^+/Δmye^ and Gpx4^Δ/Δmye^ mice	ROS released by bone marrow cells	Myeloid cell-derived ROS induce epithelial mutagenesis.	Lack of Gpx4 in bone marrow cells leads to increased ROS production, while increased oxidative stress can induce tumor development.	[55]
Colorectal cancer	Human samples, dextran sodium sulfate induced colitis in mice, and acute mucosal injury in mice	Pro-inflammatory particles miR-23a and miR-155	Neutrophils induce the impairment of colon healing and genomic instability.	Neutrophil-derived miR-23a and miR-155 induce double-strand breaks, leading to the occurrence of cancer.	[56]
Lung adenocarcinoma	A549 (K-ras mutant) and K-ras WT 201 T cell lines and LSL-K-ras mice and cell lines	NE	NE derived from neutrophils promotes lung tumor growth.	NE degrades IRS1, leading to activation of PI3K, which ultimately promotes cancer proliferation.	[57]
Melanoma	Human samples and RAS driven tumor formation in zebrafish	PGE_2_	Neutrophils promote cancer cell proliferation and growth by releasing nutrient factor PGE_2_.	The PGE_2_ released by neutrophils induced by wound inflammation can promote the proliferation of pretumor cells.	[58]
Prostate cancer	Human samples, Pten^pc−/−^ mice, and PC3 human prostate cancer cell lines	APOE released by tumor cells	Tumor-induced aging (TREM2^+^) neutrophils persist in the tumor microenvironment and exert immunosuppressive effects.	The APOE released by tumor cells binds to TREM2 on neutrophils, inducing neutrophil aging. A series of cytokines secreted by TREM2^+^ neutrophils mediate immune suppression.	[59]
Prostate cancer	Human samples, male Il1ra^−/−^ mice, and Pten^−/−^ mouse embryonic fibroblasts (MEFs)	IL-1RA	Tumor-infiltrating neutrophils antagonize tumor cell aging.	IL-1RA secreted by neutrophils can antagonize aging.	[60]
Breast cancer	MDA-MB-231 cell line and T47D human breast cancer cell line	OSM	OSM secreted by neutrophils induces VEGF expression in breast cancer cells, promoting angiogenesis and invasion.	GM-CSF derived from breast cancer cells stimulates neutrophils to release OSM, and the latter induces VEGF expression by activating the JAK-STAT pathway after combining with tumor cells.	[61]
Colorectal cancer with liver metastasis	Human samples and intrasplenic injection of human colorectal cancer cells into mice	FGF2	Neutrophils induce metastatic angiogenesis by promoting the production of FGF2.	FGF2 can be produced directly by tumor-associated neutrophils or released by neutrophils secreting heparinase to degrade ECM.	[62]
Gastric cancer	Human gastric cancer cell lines BGC-823, MGC80–3, SGC-7901, and HGC-27	HMGB1	Exosomes derived from gastric cancer cells carrying HMGB1 induce neutrophil autophagy.	Gastric cancer cell-derived exosomes (HMGB1) induce neutrophil autophagy through the activation of the HMGB1/TLR4/NF-κB pathway, leading to the release of pro-tumor cell migration factors such as IL-1β and OSM.	[63]
Breast cancer	Murine D2.0R and human MCF-7 cell lines	NE and MMP-9	Under the induction of inflammation, NETs induce the recovery of dormant cancer cells.	NE and MMP-9 released by NETs sequentially cut laminin to produce integrin α3β1 activation epitopes.	[64]
Melanoma	Inject A375M, 1205 Lu, C8161.Cl9 or UACC 903 M cells into mice	IL-8	IL-8 secreted by melanoma promotes neutrophil recruitment and interaction with tumor cells.	β2 integrins on neutrophils and intercellular adhesion molecule-1 on tumor cells mediate their interactions.	[65]
Breast cancer, lung cancer, and colon cancer	Human samples and 4 T1, LLC, HT29, CT26 tumor-bearing mice	CXCR1 CXCR2	The barrier of NETs is beneficial for tumor cells to avoid immune toxicity damage mediated by CD8^+^ T cells and NK cells.	Tumor-derived CXCR1 and CXCR2 induce the formation of NETs, which increase the interception of circulating tumor cells.	[66]
Breast cancer	Spontaneous, experimental breast cancer metastasis in mice	Resident mesenchymal cells (MCs) in the lungs	Under the stimulation of lung-resident MC, neutrophils accumulate a large amount of lipids to provide energy for tumor cells.	MCs trigger lipid storage in neutrophils, which then transfer their stored lipids to disseminated tumor cells for survival by releasing vesicles.	[67]
Lymphoma, colon cancer, and lung cancer	EL4 lymphoma, and CT26 (colon cancer) and LLC tumor-bearing mice	MPO	Neutrophils limit antigen cross presentation of dendritic cells (DCs).	The MPO of neutrophils drives lipid peroxidation, and the subsequent transfer of this oxidized lipid from neutrophils to DC limits the antigen cross-presentation effect of the latter.	[68]
Lewis lung carcinoma, lymphoma, colon carcinoma, and sarcoma	GEM mice for LLC, CT26, and KPC, EL-4 tumor-bearing mice, and the EL4, LLC, CT26, and TC-1 F244 cell lines	FATP2 and PGE2	Neutrophils expressing FATP2 mediate immune suppression by inducing PGE2 synthesis.	The expression of FATP2 by neutrophils leads to an increase in the uptake of arachidonic acid, thus promoting the biosynthesis of PGE2 and ultimately exerting T cell inhibition.	[25]
Lymphoma, Lewis lung carcinoma, and colon carcinoma	Human samples and EL4, EG7, LLC, CT26, and MC-38 tumor-bearing mice	Ferroptosis and PGE2	Neutrophils undergoing ferroptosis induce tumor immunosuppression.	The number of neutrophils undergoing ferroptosis decrease, but lipid mediators such as PGE2 limit the activity of T cells.	[69]
Lewis lung carcinoma	PL and LLC tumor-bearing mice	ARG1	ARG1 limits the function of T cells by consuming arginine, and its expression is predominant in neutrophils.	Neutrophils actively transcribe ARG1 through the membrane-associated protein A2/TLR2/MYD88 axis, promoting the immunosuppressive effect of neutrophils.	[70]
Hepatocellular carcinoma	Human samples and H22 tumor-bearing mice	TNF-α, GM-SCF, and the PD-L1/PD-1 axis	PD-L1 expression on neutrophils negatively regulates adaptive immune T cells.	Tumor-derived cytokines such as TNF-α and GM-SCF help induce PD-L1 expression on neutrophils.	[71]
Hepatocellular carcinoma	Human samples	IL-6, PD-L1/PD-1 axis	CAFs promote immune suppression of cancer cells through the IL6-STAT3-PDL1 signaling pathway.	IL-6, secreted by HCC-derived CAF, induced the upregulation of PD-L1 expression in neutrophils through the JAK-STAT3 signaling pathway.	[72]
Gastric cancer	Human samples and the human BGC-823 cell line	HMGB1 and the PD-L1/PD-1 axis	Gastric cancer cells activate the immunosuppressive effect of neutrophils through the GM-CSF-PD-L1 pathway.	Tumor cell-derived vesicle transport (HMGB1) upregulates PD-L1 expression in neutrophils by activating the STAT3 pathway.	[73]
Melanoma, Lewis lung carcinoma, colon carcinoma, and mammary carcinoma	B16-F10, LLC, CT26, and 4 T1 tumor-bearing mice	Hypoxia and the PD-L1/PD-1 axis	Hypoxia selectively upregulates PD-L1 on neutrophils, affecting T cell antitumor activity.	Hypoxia-induced HIF-1α directly binds to the proximal HRE of PD-L1 and upregulates the expression of PD-L1 on neutrophils.	[74]
Burkitt lymphoma, breast cancer, colon adenocarcinoma, and renal carcinoma	Raji, SK-BR-3, DLD-1, LS-174 T, HT-29, HCT-116, Caki-1, RCC4, RCC10, and TK10 cell lines and SRG mice	CD47/SIRPα axis	Tumors impair the antitumor activity of neutrophils and macrophages through the CD47/SIRPα axis.	Tumors express the “don’t eat me” signal CD47, which when combined with the ligand SIRPα on macrophages and neutrophils causes tumors to evade immune injury.	[75]
Cervical cancer	TC-1 tumor-bearing mice	C5a, ROS and RNS	C5a induces neutrophil disruption of T cell activity.	C5a promotes the migration of neutrophils to tumors and the release of ROS and RNS from neutrophils to impair T cell activity.	[76]
Breast cancer	Metastatic C4 T1 mice	IL-10 and IL-12	Neutrophils induce macrophage polarization toward the M2 phenotype, promoting the formation of an immunosuppressive environment.	The production of IL-10 by neutrophils leads to a decrease in IL-12 production by macrophages, ultimately leading to polarization to the M2 phenotype.	[77]
Lung adenocarcinoma	Autochthonous GEM mice	Commensal Microbiota, γδ T cells and IL-17	The symbiotic microbiome induces γδ T cells and neutrophils to copromote tumor progression.	The microbiota stimulates the production of IL-1β and IL-23 by neutrophils, which induce the activation of lung-resident gamma-δ T cells. γδ T cells, in turn, produce IL-17 to induce neutrophil infiltration.	[78]

### Neutrophils promote the initiation and proliferation of local tumors

Chronic inflammation and genetic instability caused by neutrophils promote cancer development. In infected mice, neutrophils were observed to gather and release high levels of NO in the colon, speeding up intestinal inflammation and cancer formation [54]. Additionally, neutrophils release harmful substances that damage DNA and heighten cancer risk. In early intestinal tumor stages, ROS from bone marrow cells cause DNA harm, aiding carcinogenic changes [55]. Independent of the ROS mechanism, neutrophil-derived particles with proinflammatory microRNAs (miR-23a and miR-155) also contribute to double-strand breaks in intestinal epithelial cells [56]. Interestingly, depleting the entire neutrophil population using Ly6G antibodies weakens the carcinogenic effects in chemically induced and spontaneous cancer models, underscoring the significant potential of neutrophils in carcinogenesis.

**Fig. 4:**
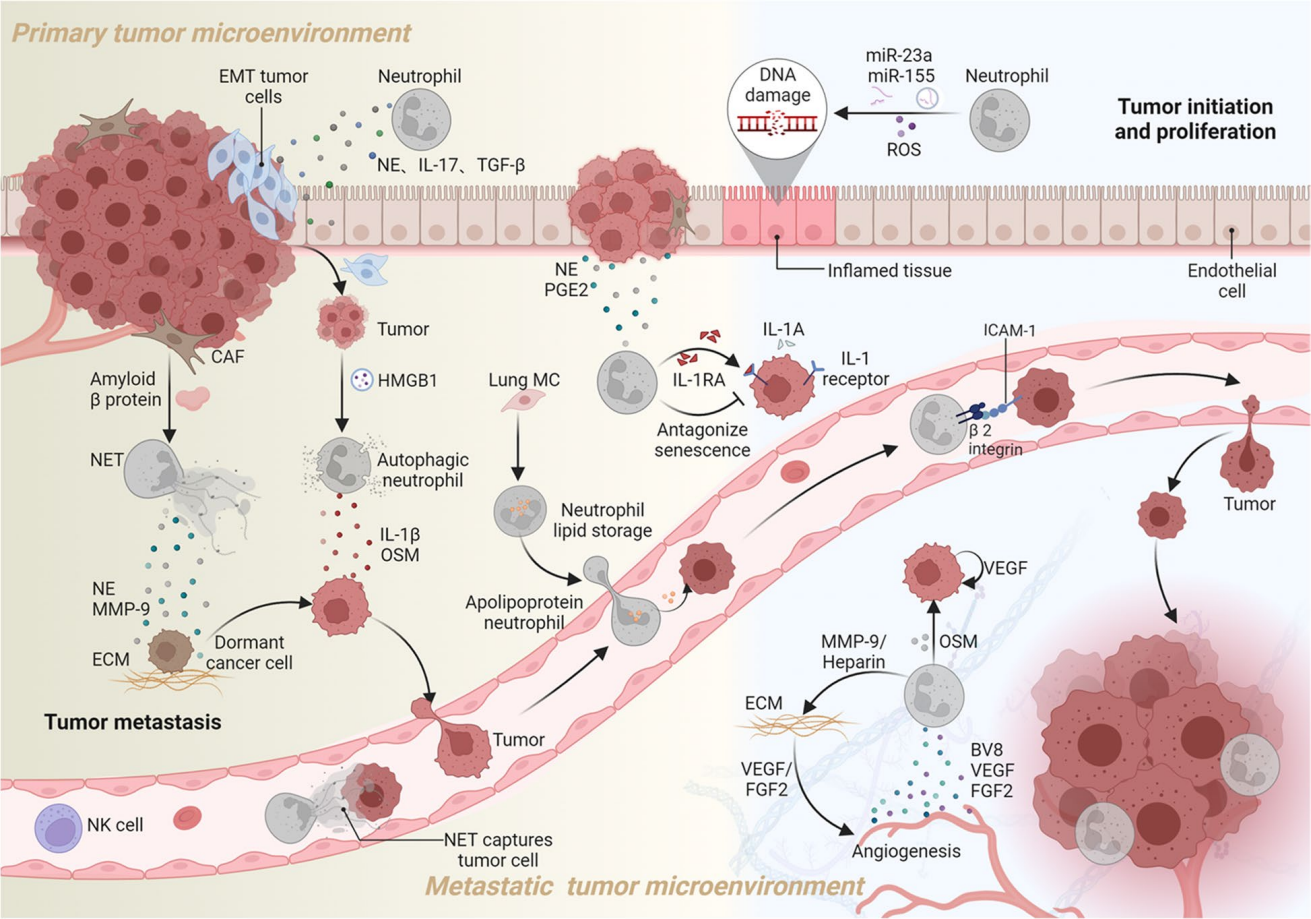
Neutrophils promote tumor initiation, proliferation, metastasis, and angiogenesis. Neutrophils promote tumor progression by releasing proinflammatory particles (miR-23a, miR-155) and reactive ROS, which cause DNA damage and support carcinogenesis. They contribute to tumor proliferation through the release of NE and prostaglandin E2 (PGE2). Neutrophils also secrete interleukin-1 receptor antagonist (IL-1RA) to protect tumors from senescence and secrete IL-17, TGF-β, and NE, promoting epithelial-mesenchymal transition (EMT) and facilitating metastasis. Tumor-derived HMGB1 exosomes induce neutrophil autophagy, leading to the release of IL-1β and oncostatin M (OSM), which promote tumor migration. Amyloid β from cancer-associated fibroblasts (CAFs) stimulates the formation of NETs, releasing NE and MMP-9 to degrade the extracellular matrix (ECM) and revive dormant cancer cells. Lung mesenchymal cells (MCs) trigger lipid stores in neutrophils, providing nutrients to disseminated tumor cells. NETs also trap circulating tumor cells, protecting them from immunotoxic effects mediated by natural killer (NK) cells. Interactions between neutrophils (via β2 integrin) and tumor cells (through intercellular adhesion molecule-1) enable tumor evasion from blood shear. Additionally, OSM released by neutrophils induces tumor cells to secrete vascular endothelial growth factor (VEGF), promoting angiogenesis. Neutrophils themselves release Bv8, VEGF, and liver fibroblast growth factor 2 (FGF2), directly contributing to tumor angiogenesis. Neutrophil-derived MMP-9 and heparin degrade the ECM, releasing VEGF and FGF2, further supporting tumor angiogenesis

During tumor progression, mediators derived from neutrophils directly or indirectly facilitate tumor growth. NE derived from neutrophils can degrade insulin receptor substrate 1 (IRS1), a negative regulator, leading to the activation of phosphoinositide 3-kinase (PI3K) and promoting the proliferation of lung cancer cells both in vivo and in vitro [57]. In a zebrafish model driven by RAS for tumor formation, neutrophils promoted cancer cell proliferation and growth by releasing PGE_2_ [58]. In a mouse model of prostate cancer, tumor cell-released apolipoprotein E (APOE) interacts with triggering receptor expressed on myeloid cells 2 (TREM2) on neutrophils, triggering the senescence process in neutrophils [59]. Nevertheless, such senescent-like neutrophils generate a spectrum of bioactive molecules collectively referred to as the senescence-associated secretory phenotype (SASP), can create an immunosuppressive environment and inhibit the activation of NK cells and cytotoxic T lymphocytes, fostering chronic inflammation and driving tumor progression [79]. Tumor-infiltrating neutrophils not only regulate their own senescence but also shield proliferating tumor cells from senescence, leading to sustained tumor cell growth. This antagonistic senescence response is attributed to the release of interleukin-1 receptor antagonist (IL-1RA) by neutrophils [60] (see Fig. 4 for illustration).

Prolonged chronic inflammation and the resulting tissue damage create a favorable environment for tumor formation, leading to tumors being referred to as “nonhealing wounds.” However, the inflammatory response of neutrophils in the development of tumors still needs further investigation.

### Neutrophils promote tumor angiogenesis

Angiogenesis is critical for tumor growth and metastasis, as it supplies nutrients and eliminates metabolites [80]. During tumor development, neutrophils release Bv8 and vascular endothelial growth factor (VEGF), effectively promoting tumor angiogenesis [81, 82]. Moreover, neutrophil-secreted MMP-9 degrades the extracellular matrix (ECM), releasing VEGF [83]. When exposed to GM-CSF from breast cancer cells, neutrophils release large amounts of oncostatin M (OSM), which induces VEGF expression through activation of the Janus-activated kinase/signal transducer and activator of transcription (JAK-STAT) pathway in cancer cells [61]. However, in VEGF-induced tumor models, liver fibroblast growth factor 2 (FGF2) can compensate for angiogenesis [84]. FGF2, found in neutrophils and the ECM in liver metastases, can be released by neutrophil-secreted heparanase, promoting angiogenesis [62]. Therefore, Neutrophils seem to produce FGF2 directly and trigger its release from the ECM (Fig. 4). Chemokines containing the ELR tripeptide motif (CXCL1–3, CXCL5–8) promote angiogenesis, and those lacking this motif (CXCL4, CXCL9–11, and CXCL14) inhibit angiogenesis (see review [85, 86]). The ELR^+^ subset may promote angiogenesis by directly binding to the receptor CXCR2 expressed on tumor blood vessels or indirectly recruiting leukocyte subsets. Thus, the balance between ELR^+^ and ELR^−^ chemokines is crucial for regulating tumor angiogenesis.

Overall, Neutrophils can boost proangiogenic factors, making patients less responsive to antiangiogenic treatments. In addition, Antiangiogenic therapy-induced hypoxia triggers mechanisms of drug resistance in tumors, such as promoting invasion and metastasis, coopting the normal vascular system for oxygen and nutrients (co-selection of blood vessels), and the transformation of tumor cells into vascular-like or endothelial-like cells involved in angiogenesis (vascular mimicry) [87–89]. Therefore, further research is necessary to explore the potential mechanisms underlying angiogenesis or its substitution.

### Neutrophils promote tumor metastasis

Neutrophils play crucial roles in promoting tumor migration, formation of the pre-metastatic niche and awakening dormant cancer cells in distant sites. Epithelial-mesenchymal transition (EMT) decreases cell adhesion, aiding tumor cells in migrating from the primary site to distant areas [90]. Neutrophils secrete mediators such as IL-17, TGF-β, and NE, all of which bolster tumor EMT [91–93]. Exosome transporter high-mobility group box 1 (HMGB1) derived from gastric cancer cells induces neutrophil autophagy via the HMGB1/Toll-like receptor 4 (TLR4)/nuclear factor-κB (NF-κB) signaling pathway, releasing pro-tumor cell migration factors, such as IL-1β and OSM [63]. Even after successful removal of primary tumors, patients may face renewed cancer spread years later due to dormant cancer cells [94]. In mice model of breast cancer, persistent lung inflammation was found to trigger the transformation of dormant cancer cells into invasive metastatic tumors [64]. Mechanistically, inflammation induces NE and MMP-9 release during NET formation. These enzymes cleave adhesion proteins, generating integrin α3β1-activated epitopes that revive dormant cancer cells [64]. Furthermore, amyloid protein β secreted by cancer-associated fibroblasts (CAFs) also drives the formation of NETs, supporting tumor development [95].

Neutrophils support cancer metastasis by acting as adhesion substrates and energy suppliers. Tumor cells must withstand immune attacks and blood flow shear forces to survive when spreading to distant locations [96, 97]. For breast cancer patients, high levels of circulating tumor cell-neutrophil clusters indicate a greater risk of metastasis [98]. Melanoma cells release IL-8, attracting neutrophils to bind with cancer cells. This process involves β2 integrins on neutrophils and intercellular adhesion molecule-1 on tumor cells, which stabilize the tumor cells and enhance their spread to the lungs [65]. Moreover, NETs trap circulating tumor cells, helping them evade immune cell attacks [66]. Neutrophils also provide energy for metastatic tumor cells. In a breast cancer study, lung mesenchymal cells (MCs) prompted neutrophils to store lipids that could be transferred to tumor cells, supporting their growth and survival, leading to lung metastasis [67] (Fig. 4).

Therefore, neutrophils are pivotal in all phases of tumor spread. Interestingly, organs where metastases occur are not just passive targets; they are actively chosen and modified by the primary tumor before spreading [99]. Common sites for metastasis include the lungs, liver, brain, bones, and lymph nodes, with specific integrins in tumor-derived exosomes guiding organ selection. For example, α6β4 and α6β1 integrins in exosomes are linked to lung metastasis, while αvβ5 integrin is associated with liver metastasis [100]. The precise link between specific exosomes and various metastatic organs is still under active investigation.

### Neutrophils regulate other immune cells to drive protumor immune responses

Metabolic abnormalities within the TME, such as abnormal lactate metabolism, lipid metabolism, and amino acid metabolism, significantly impact the antitumor activity of immune cells [69, 101, 102]. Tumors compete with T cells for glucose uptake, leading to T cell depletion and immune evasion [103]. Tumor cells relying on glycolysis produce lactic acid, shaping neutrophils and macrophages towards a pro-tumor state, creating immune-suppressing surroundings [101, 104]. During the development of tumors, lipids are not only energy sources of tumor cells and structural components of membranes but also alter the metabolic crosstalk between different immune cells in the TME [105, 106]. Neutrophil derived myeloperoxidase (MPO) induces lipid peroxidation, hindering DCs’ antigen presentation to CD8^+^ T cells [68]. Neutrophils expressing FATP2 absorb arachidonic acid, boosting PGE2 production that hampers T cell function [25]. Additionally, in the TME, neutrophils undergoing ferroptosis release lipid mediators, such as PGE2, that limit T cell activity [69]. Tumor-activated neutrophils release arginase 1 (ARG1), reducing L-arginine levels and impacting T cell function [102]. Recent research has shown that neutrophil lineage cells, rather than non-monocytes or macrophages, are the primary sources of ARG1 expression in human non-small cell lung cancer [70].

Neutrophils in the tumor microenvironment induce autoimmune checkpoint ligand expression, exerting immunosuppressive effects. Tumor-driven factors like TNF-α, GM-CSF, HMGB1, IL-6, CCL20, and hypoxia boost PD-L1 levels in neutrophils, dampening immune responses, particularly T cells [71–74, 107–109]. For instance, GM-CSF from gastric cancer, HMGB1 via extracellular vesicles, and IL-6 from HCC-associated CAFs enhance PD-L1 expression in neutrophils through JAK-STAT3 signaling [72, 73, 109]. Moreover, both tumors and healthy tissues express CD47, a “don’t eat me” signal, binding to SIRP receptors on macrophages and neutrophils, aiding tumors in evading immune eradication [75].

**Fig. 5:**
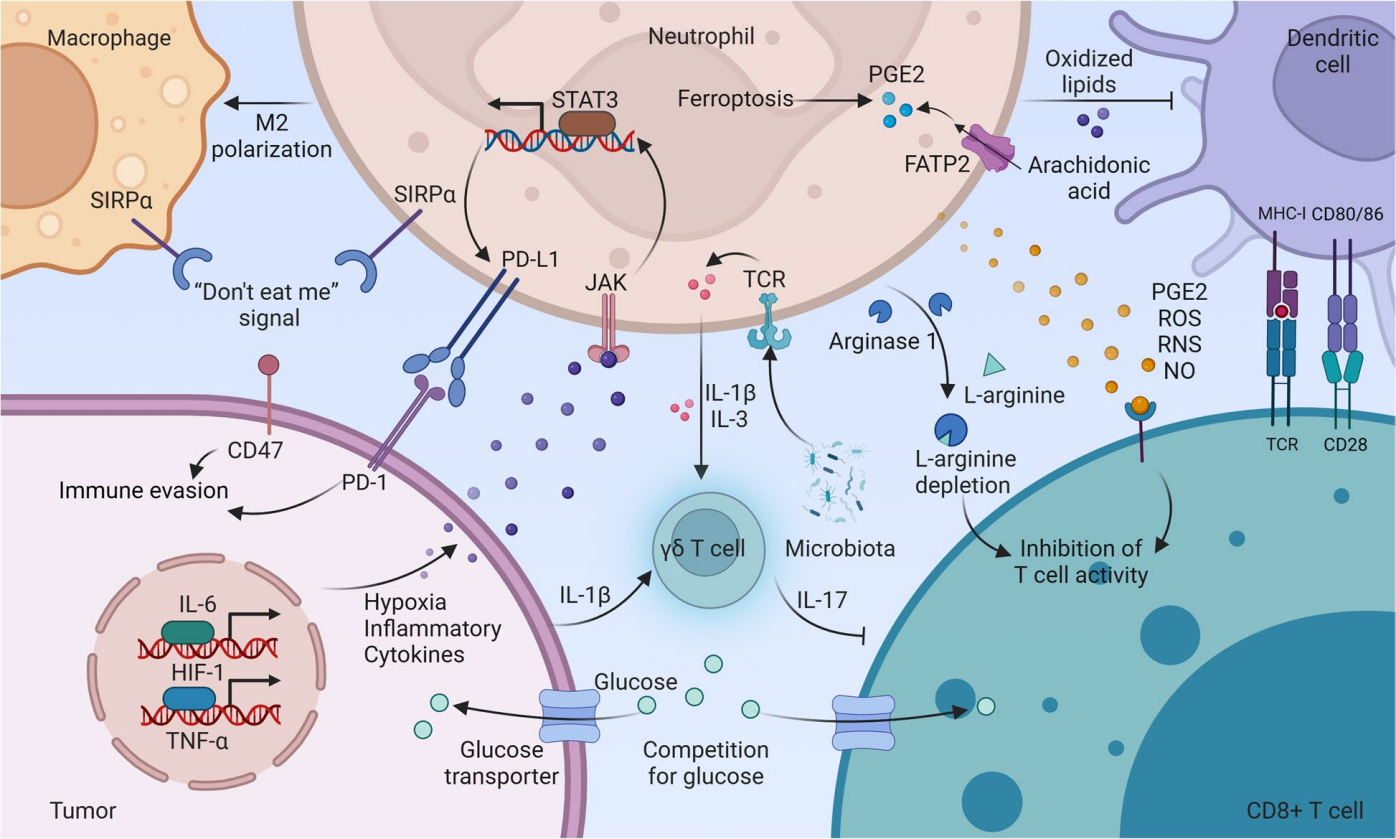
Neutrophils regulate immune cells to drive protumor immune responses. Neutrophil-produced IL-10 promotes M2 polarization in macrophages. Tumors evade immune attack through CD47, a “don’t eat me” signal that interacts with SIRPα on macrophages and neutrophils. Tumor-derived mediators (TNF-α, IL-6, GM-CSF, HMGB1, and CCL20) and hypoxia activate JAK-STAT3, upregulating PD-L1 in neutrophils. Microbiota-stimulated neutrophils secrete IL-1β and IL-3, along with tumor-derived IL-1β, activating γδ T cells. Activated γδ T cells produce IL-17, recruiting neutrophils to suppress CD8 T cells. Neutrophils expressing FATP2 enhance PGE2 biosynthesis by promoting arachidonic acid uptake. Neutrophils undergoing ferroptosis release lipid mediators, including PGE2. PGE2, ROS, RNS, and NO released by neutrophils limit T cell activity. Neutrophil-derived MPO drives lipid peroxidation, impairing dendritic cell antigenic cross-presentation. Neutrophil-released ARG1 reduces L-arginine availability, limiting T cell function. Tumors compete with T cells for glucose uptake, depleting T cells and evading the immune response

Neutrophils influence other immune cells through cytokine secretion, hindering anti-tumor responses. In mice, the complement factor C5a drives neutrophil migration to tumors, releasing ROS and reactive nitrogen species (RNS) that hamper T cell function [76]. Altering TCR recognition and reducing chemokine receptor expression, along with nitrosylation of tyrosine, obstruct T cell movement towards tumors, creating an RNS-based barrier [76, 110]. The RNS-induced immune “cold” environment orchestrates the preferential entry of marrow cells with immunosuppressive characteristics, compromising cancer immunotherapy for various solid tumors, including pancreatic ductal adenocarcinoma [111]. Clearing RNS could enhance current treatment effectiveness. Immunosuppressive neutrophils producing IL-10 lower IL-12 from macrophages, switching them from M1 to M2 [77]. Microbiota TLR signals prompt neutrophils to release IL-1β and IL-23, activating lung-resident γδ T cells [78]. Nevertheless, IL-17 from γδ T cells recruits neutrophils, suppressing CD8^+^ T cell action in breast tumors [112]. This reciprocal interaction between γδ T cells and neutrophils forms amplification loops within the tumor microenvironment, effectively promoting tumor progression (Fig. 5).

Thus, neutrophils shape the immune system to aid tumor development, particularly impacting T cells. Essentially, neutrophils assist tumors in evading immune monitoring by changing metabolism, adjusting immune checkpoints, and modulating immune cell subsets via cytokines.

## Are neutrophils friends or foes during cancer progression?

**Fig. 6:**
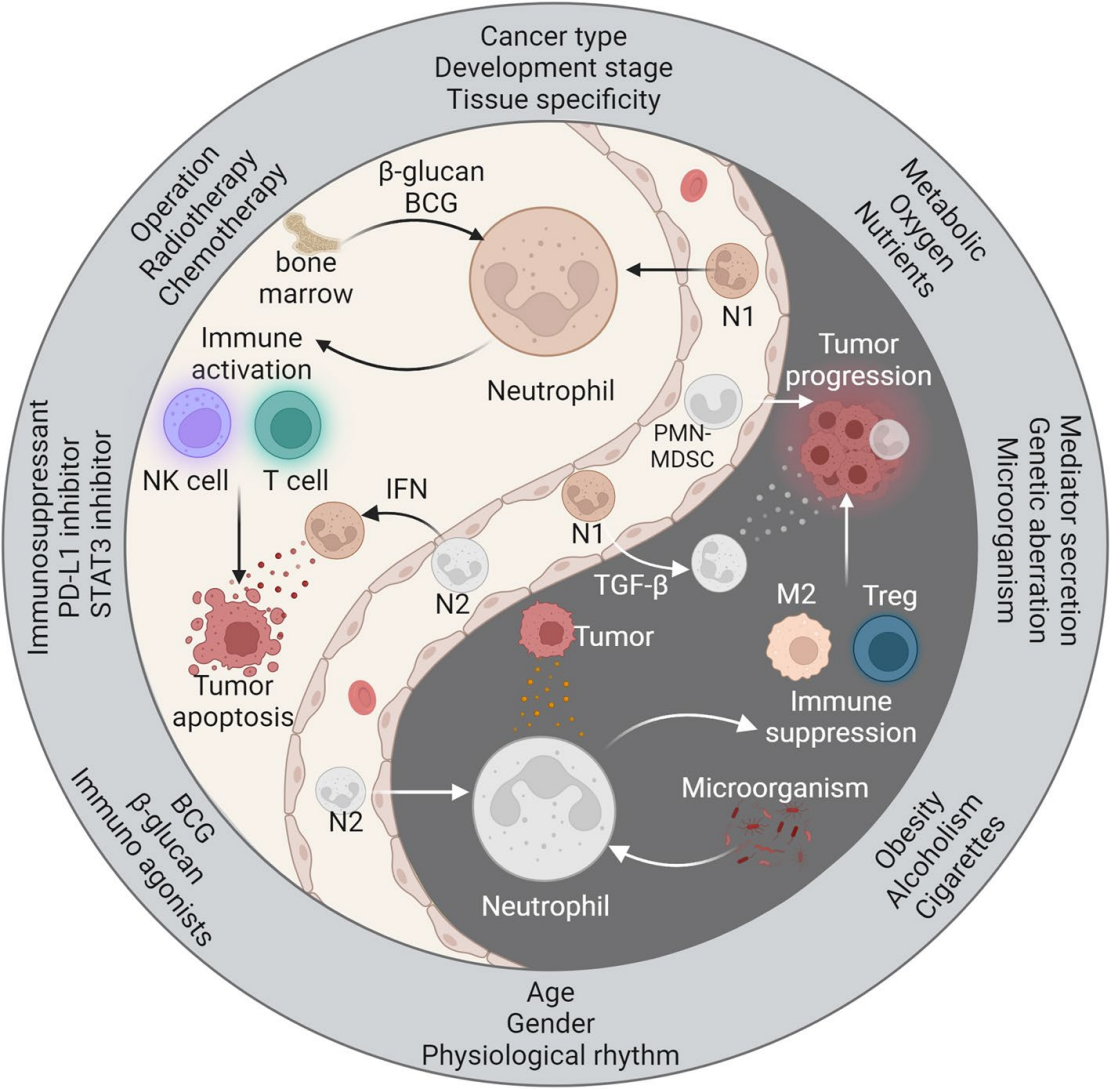
The Yin and Yang profiles of neutrophils in the progression of cancer. β-glucan and BCG stimulate neutrophils to develop antitumor innate immune memory in the bone marrow. Antitumor neutrophils (N1) induce tumor apoptosis by activating T cells and NK cells and releasing cytokines. However, tumor-derived factors and microbes can influence neutrophils to adopt a protumorigenic phenotype (N2). N2 neutrophils contribute to an immunosuppressive microenvironment by recruiting M2-type macrophages and Tregs. They also secrete mediators that directly promote tumor progression. IFN and TGF-β drive the formation of N1 and N2 neutrophil phenotypes, respectively. Moreover, neutrophil polarization varies based on tumor characteristics and patient status. Discontinuation of surgery, chemotherapy, and radiotherapy can lead to neutrophil-induced tumor recurrence. Adjuvant therapy using immunoagonists (e.g., β-glucan, BCG vaccine) and immunosuppressants (e.g., PD-L1, STAT3 inhibitors) enhances the antitumor effect of neutrophils. Poor patient habits, endogenous factors, and tumor metabolic reprogramming influence the tumor-promoting effect of neutrophils

Neutrophils exhibit a dual role in the tumor microenvironment, with both antitumor and protumor effects. On the one hand, neutrophils serve as immune system defenders and exert inhibitory effects on tumors. However, under the influence of the tumor microenvironment, the release of immature neutrophils or phenotypic changes in neutrophils promotes tumor growth. The intricate relationship between neutrophils and cancer progression depends on multiple factors, such as tumor characteristics, endogenous influences, exogenous therapeutic interventions, and the overall health status of patients (Fig. 6).

The characteristics of tumors within the host, including cancer type, developmental stage, tissue specificity, and metabolic reprogramming, closely influence the phenotypic state of neutrophils [51, 113–117]. The role of neutrophils in cancer is highly intricate, and there may be variations across different cancer types. In most cancer patients, high levels of TAN are generally associated with poorer treatment outcomes. However, in colorectal cancer patients, TAN infiltration is linked to a favorable prognosis [113–115]. We hypothesize that different cancers may exhibit varying levels of inflammation, leading to differences in the recruitment and infiltration of neutrophils into tumor tissue. The developmental stage of cancer, whether early or late, significantly impacts the phenotype and characteristics of neutrophils. For instance, LNs are crucial sites for initiating adaptive immune responses and serve as important locations for tumor cell metastasis [51]. In the early N0 stage, neutrophil infiltration is associated with improved patient survival. Conversely, in the late N1–3 stage, a high presence of neutrophils indicates a decrease in the patient survival rate [51]. The phenotypic transformation of neutrophils may be attributed to changes in the immune microenvironment at different developmental stages. Additionally, neutrophils exhibit distinct functional characteristics in different target tissues within the context of cancer. Neutrophils in the lungs and intestines demonstrate significant angiogenic properties, while they are nearly absent in the intestines and skin under sterile conditions [116]. Metabolic reprogramming plays a beneficial role in regulating energy metabolism and facilitating rapid cell growth and proliferation and is considered an emerging marker of cancer [69, 101]. However, the competitive consumption of abundant oxygen and nutrients by cancer cells typically impairs the metabolic adaptability of immune cells infiltrating tumors [103, 118]. Concurrently, abnormal metabolites or intermediates in tumors ultimately influence the effects and functions of immune cells, thereby promoting immune evasion within the tumor microenvironment [119]. When tumors consume glucose, they generate a substantial amount of lactic acid, leading to the formation of an acidic extracellular tumor microenvironment that hampers the antitumor effects of various immune cells [101, 104]. Furthermore, the establishment of a hypoxic environment promotes the upregulation of PD-L1 in neutrophils [74]. Thus, the presence of high levels of lactic acid, low pH, and hypoxia in the tumor microenvironment exacerbates the negative impact on immune cell functionality.

Endogenous factors such as mediator secretion, genetic aberrations, and microorganisms play crucial regulatory roles in neutrophils [120–122]. Mediators derived from cancer cells and their surrounding stromal cells, such as IL-6, PGE2, TGF-β, hyaluronic acid fragments (HA), and G-CSF/GM-CSF, contribute to the reprogramming of neutrophils into a tumor-promoting phenotype [16, 25, 120, 123, 124]. CircPACRGL, derived from colorectal cancer (CRC), is a circRNA that increases TGF-β expression upon uptake of miR-142-3P or miR-506-3P, leading to the polarization of neutrophils to the N2 phenotype and eventual tumor progression [125]. Conversely, IFN-γ (type II IFN) induces IRF1 expression, which is essential for the tumor-suppressive effect of neutrophils in the context of successful immunotherapy [52]. Moreover, genetic aberrations in the tumor itself alter the cytokine secretion profile, thereby inducing the accumulation of immunosuppressive neutrophils. In a mouse model with Tp53-deficient tumors, WNT ligands secreted by cancer cells induced IL-1β secretion by tumor-associated macrophages, ultimately leading to the aggregation of immunosuppressive neutrophils [121]. Unexpectedly, microorganisms play essential roles in regulating the functional heterogeneity of neutrophils. Local microorganisms not only prolong the lifespan of neutrophils under inflammatory conditions but also promote neutrophil-mediated inflammation and tumor growth [78]. For example, CRC-derived *Escherichia coli* breaks the intestinal vascular barrier and translocates to the liver, further promoting the accumulation of neutrophils and leading to the formation of an ecological niche before tumor metastasis [122]. The regulatory effects of various endogenous factors on neutrophils are complex and diverse, playing an important role in regulating neutrophil function and inflammatory immunity.

Exogenous therapeutic interventions, including surgery, chemotherapy/radiotherapy, immune agonists (such as β-glucan and BCG), and immunosuppressants, also modulate the functional properties of neutrophils [41, 126–130]. The surgical process may lead to the shedding and dissemination of tumor cells, ultimately resulting in cancer recurrence in postoperative patients [131]. However, surgical trauma-induced inflammatory reactions activate neutrophils and create a tumor microenvironment conducive to tumor growth and spread [126]. Moreover, surgical stress often triggers the formation of NETs, which act as protective barriers, facilitating the metastasis of tumor cells to distant sites [66]. Therefore, precise resection or postoperative adjuvant therapy helps eliminate tumor recurrence. Radiation exposure induces acute lung injury and promotes neutrophilic pro-metastatic responses, enhancing the colonization of tumors at distant sites [127]. Disseminated tumors after chemotherapy cessation induce neutrophil recruitment into the metastatic site of the liver and promote increased metastatic growth [128]. Mechanistically, growth arrest-specific 6 (Gas6) expressed by neutrophils activates AXL receptors on tumor cells, promoting their regeneration [128]. Hence, combining chemotherapy with the inhibition of the Gas6/AXL signaling axis may be effective in reducing cancer recurrence. In fact, β-glucan and BCG vaccines act as agonists of cellular immunity, inducing neutrophils to form antitumor innate immunological memory at the bone marrow level. β-glucan-trained granulocytes possess the ability to limit tumor growth, and this immunological trait is retained at the bone marrow level and can be transferred to untrained recipient mice [41]. BCG vaccination not only promotes the generation of bone marrow lineages biased toward granulocytes but also induces the long-term functional transformation of circulating neutrophils, resulting in enhanced antibacterial function of neutrophils under the co- stimulation of multiple pathogens in vitro [129, 132]. Currently, the BCG vaccine is used as a first-line adjuvant treatment for bladder cancer [133]. Furthermore, targeting key immune checkpoints and immunosuppressive molecules enhances the antitumor activity of neutrophils. Activation of the STAT3 signaling pathway typically leads to the upregulation of PD-L1 in neutrophils, enabling tumors to evade neutrophil surveillance and clearance [73, 109]. Therefore, the development of targeted inhibitors of PD-L1 and STAT3 would be beneficial in restoring the antitumor function of neutrophils.

The state of patients can vary with age, sex, physiological rhythm, and obesity, which have diverse effects on immune cell function [134–139]. Generally, immune function tends to gradually decline with age, which may explain why elderly individuals are more susceptible to infections, cancer, and immune-related diseases [140]. During the aging process, dysfunction of neutrophils occurs due to weakened signal transduction of specific receptors [134]. Additionally, young women exhibit higher levels of mature neutrophils and stronger activation responses than do young men [135]. In pregnant women, the frequency of immature neutrophils in the blood is higher than that in healthy women, and these immature neutrophils may play beneficial roles in maintaining maternal-fetal tolerance [135, 141]. In the process of melanoma-induced metastasis, a clear diurnal pattern has been observed, where many metastatic tumors appear in the lungs during cell injection in the morning, with an almost nonexistence of metastatic lesions during injection at night [136]. This pattern aligns with the circadian rhythm of neutrophil aging [34]. Notably, the depletion of neutrophils leads to the alleviation of tumor metastasis, providing new evidence for targeted neutrophil immunotherapy and time-controlled drug delivery [136]. Obesity and alcoholism exacerbate increases in CXCL1, resulting in the increased infiltration of neutrophils into the liver and synergistically promoting the occurrence of steatohepatitis [139]. In an obese mouse model, adipose tissue was shown to induce an increase in lung neutrophils, thereby facilitating cancer cell metastasis [137]. Smoking exposure is recognized as a risk factor for various cancers. The inflammatory response triggered by smoking induces changes in the number and function of neutrophils, implicating them as potential participants in cancer progression [138]. Nicotine in cigarette smoke stimulates N2-neutrophil polarization through the STAT3 pathway [138]. Subsequently, these neutrophils release exosomal miR-4466, promoting the metabolic transformation of brain cancer cells and ultimately accelerating the metastasis of lung cancer to the brain [138]. Furthermore, persistent lung inflammation caused by tobacco smoke exposure promotes the formation of neutrophil extracellular traps (NETs), which can aid in the recovery of dormant cancer cells [64]. Therefore, the inflammatory response induced by cigarette smoke can enhance the tumor-promoting effect of neutrophils.

In brief, neutrophils play a dual role as both allies and adversaries in cancer development. However, comprehending the complex nature of neutrophils in cancer necessitates considering a myriad of factors. These factors encompass the intrinsic characteristics of the tumor, such as cancer type, stage of progression, tissue specificity, and metabolic alterations. Additionally, endogenous factors such as mediator secretion, genetic abnormalities, and microorganism presence, as well as exogenous therapeutic interventions such as surgery, chemotherapy, radiotherapy, immune agonists, and immunosuppressants, contribute to this intricate dynamic. Furthermore, the individual state of patients, including age, sex, physiological rhythms, obesity, alcoholism, and smoking, also impacts neutrophil behavior. There might be unidentified factors influencing the plasticity of neutrophils in terms of their transcriptional profile and functional attributes.

## Cancer immunotherapy strategies targeting neutrophils

The dual nature of neutrophils within the tumor microenvironment presents challenges for targeted therapy. To enhance treatment efficacy, we propose three strategies: 1) blocking the infiltration of neutrophils into local tumors, 2) targeting the immunosuppressive function of neutrophils, 3) improving the adjuvant anticancer efficacy of neutrophils (refer to Table 4 for details).

**Table 4:** Clinical trials of targeted neutrophil therapies for cancers

Targets	Agents	Cancer applications	Phase	NCT trial number	Treatment regimen	References
CXCR1/CXCR2	Navarixin (MK-7123)	Solid tumor	II	NCT03473925	Navarixin + Pembrolizumab	NA
	SX-682 (BKT140)	Resectable pancreatic cancer	II	NCT05604560	SX-682 + Tislelizumab	NA
	Reparixin	Metastatic triple-negative breast cancer	II	NCT02370238	Reparixin + Paclitaxel	NA
	AZD5069	Metastatic castration-resistant prostate cancer	I/II	NCT03177187	AZD5069 + Enzalutamide	NA
IL-8	BMS-986253	Hepatocellular carcinoma	II	NCT04050462	BMS-986253 + Nivolumab	NA
C5aR	IPH5401	Solid tumor	I	NCT03665129	IPH5401 + Durvalumab	NA
	TJ210001	Solid tumor	I	NCT04947033	Monotherapy	NA
CD47-SIRPα	Magrolimab	TP53 mutant acute myeloid leukemia	III	NCT04778397	Magrolimab + Azacitidine	[142]
	Lemzoparlimab	Refractory non-Hodgkin’s lymphoma	Ib	NCT03934814	Lemzoparlimab + Rituximab	[143]
	CC-95251	Relapsed and/or refractory non-Hodgkin lymphoma	I	NCT03783403	CC-95251 + Rituximab	[144]
	BI 765063	Solid tumors	I	NCT03990233	BI 765063 + BI 754091	[145]
STAT3	TTI-101	Recurrent or metastatic head and neck squamous cell carcinoma	I/II	NCT05668949	TTI-101 + Pembrolizumab	NA
	Napabucasin	Metastatic colorectal cancer	III	NCT02753127	BBI-608 + FOLFIRI (5-Fluorouracil, Leucovorin, Irinotecan)	[146]
	AZD9150 (Danvatirsen)	Advanced pancreatic cancer, non-small cell lung cancer, and mismatch repair deficient colorectal cancer	II	NCT02983578	AZD9150 + MEDI4736	NA
TGF-β	LY2157299	Locally advanced rectal adenocarcinoma	II	NCT02688712	LY2157299 + Neoadjuvant chemoradiation	[147]
	M7824 (bintrafusp alfa)	Non-small cell lung cancer	III	NCT03631706	Monotherapy	NA
	NIS793	Metastatic pancreatic ductal adenocarcinoma	III	NCT04935359	NIS793 + Nab-paclitaxel/gemcitabine	[148]
G-CSF	Efbemalenograstim alfa (F-627)	Breast cancer	III	NCT04174599	Monotherapy	[149]
IL-6	Tocilizumab	Melanoma	II	NCT03999749	Tocilizumab + Ipilimumab and Nivolumab	[150]
	Siltuximab	High-risk smoldering multiple myeloma	II	NCT01484275	Monotherapy	[151]

Targeting pathways that attract neutrophils to tumors shows potential for halting cancer advancement. CXCR1/CXCR2 receptors play a crucial role in neutrophil recruitment throughout the body, responding strongly to IL-8 [15, 152]. Thus, Blocking the IL-8 pathway and its receptors CXCR1/2 emerges as a promising cancer therapy strategy. CXCR1/CXCR2 inhibitors include Navarixin (NCT03473925), SX-682 (NCT05604560), Reparixin (NCT02370238), and AZD5069 (NCT03177187). The anti-IL-8 monoclonal antibody BMS-986253 demonstrated good safety and tolerability in a phase I study as a monotherapy for cancer patients [153]. Currently, BMS-986253 has entered phase II clinical trial (NCT04050462). The C5a receptor (C5aR) on neutrophils is a key therapeutic target. Cancer-produced C5a is a potent neutrophil attractant and contributes to an immunosuppressive tumor environment by upregulating molecules like ARG1, CTLA4, and PD-L1 [154]. Blocking C5a/C5aR signaling holds promise as an effective anti-cancer approach. Clinical trials are underway to assess C5aR antagonists’ efficacy in cancer treatment, including IPH5401 (NCT03665129) and TJ210001 (NCT04947033).

Adjusting the immune-dampening signals of neutrophils in the tumor setting to revive their natural anticancer role holds significant promise. One approach is to target critical immune checkpoints and suppressive molecules to bolster neutrophils’ anticancer functions. Blocking the CD47-SIRPα interaction could amplify neutrophils’ anticancer impact [155]. Promising CD47 antibodies like Magrolimab and Lemzoparimab have shown early clinical benefits and good tolerability [142, 143]. The novel anti-SIRPα antibody CC-95251 (NCT03783403) and the SIRPα inhibitor BI 765063 (NCT03990233) have undergone Phase I study evaluations. Direct STAT3 activation fosters oncogene expression, spurring tumor advancement and immune suppression [72]. Thus, targeting the key regulatory protein STAT3 holds significant promise as an anticancer therapy. Currently effective STAT3 inhibitors include TTI-101, Napabucasin, AZD9150 [146, 156, 157]. TGF-β is a key cytokine in the regulation of immune function and is essential for both neutrophil recruitment and reprogramming [16]. In a phase II trial, the oral TGF-β receptor type I kinase inhibitor LY2157299, when used with neoadjuvant radiochemotherapy, improved complete remission to 32% with good tolerance [147]. M7824 (bintrafusp alfa), a dual-action fusion protein inhibiting TGF-β and PD-L1, displayed promising anticancer effects in a Phase II study [158]. M7824 (NCT03631706) and NIS793 (NCT04935359) have entered Phase III evaluation.

Cancer chemotherapy often causes bone marrow suppression and low neutrophil count, raising infection risks [159]. G-CSF, crucial for boosting neutrophil levels, is frequently used to prevent chemotherapy-induced neutropenia [159]. In a Phase III study with breast cancer patients, F-627, a third-generation long-acting G-CSF drug, lowered infection and febrile neutropenia rates [149]. Although G-CSF doesn’t directly fight cancer, it can improve treatment effectiveness when used alongside chemotherapy. Currently, F-627 has received approval in China for the treatment of chemotherapy-induced neutropenia [160]. IL-6, linked to immune-related adverse events (irAEs) from immune checkpoint blockade (ICB) therapy, is key for neutrophil actions [72, 161]. Anti-CTLA-4 therapy triggers IL-6 release and gut neutrophil buildup, causing an inflammatory response disrupting the gut microbiome balance and promoting irAEs [161]. Combining IL-6 inhibition with antibiotics has shown to reduce irAEs and enhance immune responses [161]. Thus, blocking IL-6 during ICB therapy enhances tumor immunity and relieves irAE symptoms. Tocilizumab and siltuximab are two effective clinical IL-6 inhibitors [150, 151].

In summary, though advancements have been achieved in neutrophil-targeting immunotherapies, challenges remain. Precisely targeting distinct neutrophil groups while maintaining a balance between immune regulation and protective functions is vital for optimal treatment results.

## Current challenges and where the field is going

Neutrophils in cancer treatment often lack specificity and have a short duration of actionTargeting neutrophils inadvertently affects other cells or antitumor neutrophils, disrupting the normal immune system and making patients more susceptible to adverse reactions such as infections [162]. For instance, the use of an anti-Gr1 antibody that recognizes Ly6G and Ly6C leads to the depletion of Ly6C-expressing cells such as monocytes and macrophages during treatment [163]. Neutrophil targeting is short-lived due to compensatory mechanisms in the complex immune system that counteract neutrophil blockade within the tumor, resulting in rapid recovery [164]. Anti-Ly6G specifically targets neutrophils, but its depletion triggers compensatory bone marrow release and even induces extramedullary spleen production of neutrophils, leading to a “rebound” effect [165]. Interestingly, newly generated circulating neutrophils express low levels of Ly6G on their membrane, rendering anti-Ly6G treatment inadequate and short-lived [166]. Thus, specific targeting of neutrophils is essential to enhance treatment efficacy and reduce toxic side effects.

Most studies on neutrophil mechanisms have been conducted in mouse models since neutrophils have a short lifespan [167]. Currently, mouse models for inducing tumor formation include genetic engineering models, chemical toxic drug models, implantation models, and humanized mouse models [167]. However, preclinical models often fail to fully replicate the complex immune system environment and disease progression in humans, making the translation of research results from mouse models to clinical treatment a challenging and time-consuming task [167]. Although humanized mouse models can bridge the gap between the human immune system and xenograft mice, they require advanced technical expertise [168]. In the future, it is essential to establish more effective and innovative preclinical models to predict and assess the applicability of various antitumor drugs in humans.

Responses to targeted neutrophil therapy may vary among individuals, highlighting the need for personalized treatment protocols. The effectiveness of treatment can be influenced by various factors, including tumor characteristics, endogenous and exogenous factors, and interpatient differences such as disease type and dietary habits [169]. Additionally, variations in drug metabolism abilities and immune system status can contribute to differences in treatment responses. Therefore, the development of personalized treatment plans is crucial to ease the burden of targeted therapy and improve treatment efficacy.

To optimize the efficacy of targeted neutrophil therapy, it is essential to develop precise methods that specifically target neutrophils while minimizing the impact on other cell types and reducing side effects [170]. Therefore, the identification of highly specific biomarkers for immunosuppressive neutrophils becomes crucial in promoting treatment effectiveness and accuracy [170]. NE, an essential physiological factor in neutrophil-mediated diseases, is exclusively present on the surface of active neutrophils and not in other leukocyte subpopulations [171]. Researchers have discovered that an α1-antitrypsin-derived peptide specifically binds to NE on activated neutrophils. Coating nanoparticles with this peptide enables selective anchoring to activated neutrophils for localized drug delivery [171].

Neutrophils have a dual role in tumor progression and participate in immune evasion and drug resistance in tumors. Thus, combining targeted neutrophil therapy with other treatment methods, such as chemotherapy and immunotherapy, can synergistically breakdown immune suppression and enhance antitumor immune effects [172, 173]. Chimeric antigen receptor (CAR)-T cell therapy based on neutrophils effectively improves therapeutic outcomes in solid tumors with heterogeneous antigen expression without additional immunotoxicity [174]. The multifunctional pro-inflammatory neutrophil activating protein (NAP), derived from *Helicobacter pylori*, possesses potent immune regulatory properties [174]. Using CAR-T cells carrying NAP can promote the recruitment and activation of neutrophils, leading to the eradication of antigenically heterogeneous tumors [174]. However, primary neutrophils have a short lifespan and are resistant to genome editing, limiting their use in CAR-modified immunotherapy [175]. CRISPR–Cas9 technology can be employed to knock in genes into human pluripotent stem cells, resulting in the generation of CLTX-T-CAR neutrophils that exhibit optimal antitumor activity against glioblastoma [175].

Neutrophils hold potential as carriers for delivering tumor drugs due to their high migratory ability during inflammatory responses and their ability to cross the blood–brain barrier, enhancing drug penetration and anticancer efficacy [176]. Currently, there are two main delivery strategies based on neutrophils: (1) using neutrophils as carriers and (2) using nanovesicles derived from neutrophil membranes as carriers [177]. Neutrophils loaded with paclitaxel liposomes can penetrate the brain and serve as postoperative adjuvant therapy for brain tumors [178]. Neutrophil membrane-derived nanovesicles enhance drug delivery. A nanoscale neutrophil-mimicking drug delivery system (NM-NP) was developed by coating neutrophil membranes onto the surface of polylactic acid glycolic acid nanoparticles (NPs) [179]. This process preserves the biological binding activity of neutrophils. After loading the second-generation proteasome inhibitor carfilzomib, NM-NP-based nanoparticles selectively deplete CTCs, prevent early metastasis, and inhibit the formation of metastatic niches [179]. However, there are limitations to drug delivery using neutrophils as carriers, such as the short in vitro lifespan of neutrophils, potential damage to neutrophil activity caused by drug loading, and challenges in controlling the storage and release profiles of nanoparticles within neutrophils [176].

Overall, targeting neutrophils for cancer therapy presents multiple opportunities and challenges. Current challenges include limitations in drug therapy, preclinical models and individual patient variability. Future studies aim to enhance the effectiveness of targeted therapy by optimizing targeting strategies, combining therapies, and exploring the potential of neutrophils as drug delivery carriers.

## Concluding remarks and perspectives

Neutrophils exhibit remarkable plasticity and complexity within the tumor microenvironment. As a subset of lymphocytes with phagocytic and cytotoxic functions, neutrophils can directly eliminate tumor cells by releasing various toxic substances or modulating the expression of apoptosis-related ligands. They also indirectly inhibit tumor progression by regulating immune system activation. However, when exposed to tumor-associated factors or exosomes, neutrophils undergo “functional remodeling,” acquiring biological properties that promote tumor immune escape and angiogenesis, thereby facilitating tumor growth and metastasis. Remodeled neutrophils contribute to tumor growth through various mechanisms and are closely associated with immune-related toxicity caused by immune checkpoint blockade. Therefore, inhibiting neutrophil migration into the tumor region not only enhances antitumor efficacy but also effectively mitigates the strong toxic response induced by immunotherapy.

Determining whether neutrophils act as allies or adversaries in cancer immunotherapy requires considering the combined influence of various factors. Tumor characteristics, including cancer type, stage, tissue specificity, and metabolic reprogramming, influence the dual nature of neutrophils. Endogenous factors such as altered immune status, genetic aberrations, and the microbiota favor the formation of tumor-promoting neutrophils within the tumor microenvironment. Therapeutic interventions, such as surgically induced inflammatory responses, activate immunosuppressive neutrophils, leading to tumor recurrence. However, immune agonists such as β-glucan and BCG can induce antitumor properties in neutrophils. Additionally, individual patient characteristics such as age, sex, and lifestyle further influence the immune landscape. Thus, comprehensively evaluating the complex role of neutrophils in cancer requires considering these multiple factors and exploring other unknown factors to develop personalized and comprehensive treatment strategies.

Neutrophils play a complex role in cancer immunotherapy, acting as a “double-edged sword” by participating in immune defense and immune tolerance maintenance. Targeting neutrophils in cancer treatment poses challenges due to potential immune dysfunction and adverse reactions, such as infections associated with new drugs or formulations. Therefore, a key focus of current clinical research is optimizing chemotherapy regimens to enhance neutrophil vitality while reducing toxicity. In future cancer immunotherapy, comprehensive approaches such as specific immunotherapy, combination therapy, and personalized therapy can be employed to maximize the therapeutic benefits of targeting neutrophils. Furthermore, leveraging cutting-edge technologies and platforms such as gene editing and nanotechnology enhances neutrophil activity and specificity, leading to superior efficacy. Bridging the gap between preclinical mouse models and human pathological environments, as well as developing personalized treatment plans for patients, will facilitate the advancement of the application of neutrophils in cancer treatment. Additionally, researchers should continue exploring the potential of neutrophils as drug delivery carriers in cancer treatment.

## Abbreviations

ADCC: Antibody-dependent cytotoxicity
APC: Antigen-presenting cell
APOE: Apolipoprotein E
ARG1: Arginase 1
BMAL1: Brain and muscle Arnt-like 1
C5aR: C5a receptor
CAFs: Cancer-associated fibroblasts
CAR: Chimeric antigen receptor
CCL2: CC motif chemokine ligand 2
CMP: Common myeloid progenitors
CXCR4: CXC chemokine receptor 4
DC: Dendritic cells
DD: Death domain
ECM: Extracellular matrix
FasL: Fas ligand
FATP2: Fatty acid transporter 2
FcRs: Fc receptors
FGF2: Fibroblast growth factor 2
G-CSF: Granulocyte colony-stimulating factor
GM-CSF: Granulocyte-macrophage colony stimulating factor
GMP: Granulocyte monocyte progenitors
H_2_O_2_: Hydrogen peroxide
HCC: Hepatocellular carcinoma
HDN: High-density neutrophils
HGF: Hepatocyte growth factor
HIF-1α: Hypoxia-inducible factor-1α
HMGB1: High-mobility group box 1
HSC: Hematopoietic stem cells
ICB: Immune checkpoint blockade
IFN: Interferon
IL-1RA: Interleukin-1 receptor antagonist
IL-6: Interleukin-6
iNOS: Inducible nitric oxide synthase
irAEs: Immune-related adverse events
IRF-8: Interferon-regulatory factor-8
IRS1: Insulin receptor substrate 1
LDN: Low-density neutrophils
LNs: Lymph nodes
LOX1: Low-density lipoprotein receptor 1
mAbs: Monoclonal antibodies
MCs: Mesenchymal cells
MDSC: Myeloid-derived suppressor cells
MMP-9: Matrix metalloproteinases
MYD88: Myeloid differentiation factor 88
NAP: Neutrophil activating protein
NASH: Nonalcoholic steatohepatitis
NE: Neutrophil elastase
NET: Neutrophil extracellular trap
NF-κB: Nuclear factor-κB
NPs: Nanoparticles
OSM: Oncostatin M
Phd3: Prolyl hydroxylase 3
PI3K: Phosphoinositide 3-kinase
ROS: Reactive oxygen species
S100A9: S100 calcium-binding protein A9
SASP: Senescence-associated secretory phenotype
scRNA-seq: Single-cell RNA sequencing
TAN: Tumor-associated neutrophils
TGF-β: Transforming growth factor-β
TLR4: Toll-like receptor 4
TNF-α: Tumor necrosis factor-α
TRAIL: Tumor necrosis factor-related apoptosis-inducing ligand
Tregs: Regulatory T cells
TREM2: Triggering receptor expressed on myeloid cells 2
TRPM2: Transient receptor potential cation channel, subfamily M, member 2
VCAM-1: Vascular cell adhesion molecule 1
VEGF: Vascular endothelial growth factor
